# The kinetic dose limit in room-temperature time-resolved macromolecular crystallography

**DOI:** 10.1107/S090904951105549X

**Published:** 2012-01-18

**Authors:** M. Schmidt, V. Šrajer, N. Purwar, S. Tripathi

**Affiliations:** a Department of Physics, University of Wisconsin-Milwaukee, Milwaukee, WI 53211, USA; b Center for Advanced Radiation Sources, The University of Chicago, Chicago, IL 60637, USA

**Keywords:** radiation damage, X-ray dose, room temperature, time-resolved crystallography, Laue crystallography

## Abstract

Protein X-ray structures are determined with ionizing radiation that damages the protein at high X-ray doses. As a result, diffraction patterns deteriorate with the increased absorbed dose. Several strategies such as sample freezing or scavenging of X-ray-generated free radicals are currently employed to minimize this damage. However, little is known about how the absorbed X-ray dose affects time-resolved Laue data collected at physiological temperatures where the protein is fully functional in the crystal, and how the kinetic analysis of such data depends on the absorbed dose. Here, direct evidence for the impact of radiation damage on the function of a protein is presented using time-resolved macromolecular crystallography. The effect of radiation damage on the kinetic analysis of time-resolved X-ray data is also explored.

## Abstract 2

The effect of the X-ray dose on room-temperature time-resolved Laue data is discussed.

## 1. Introduction   

**Figure 1:**
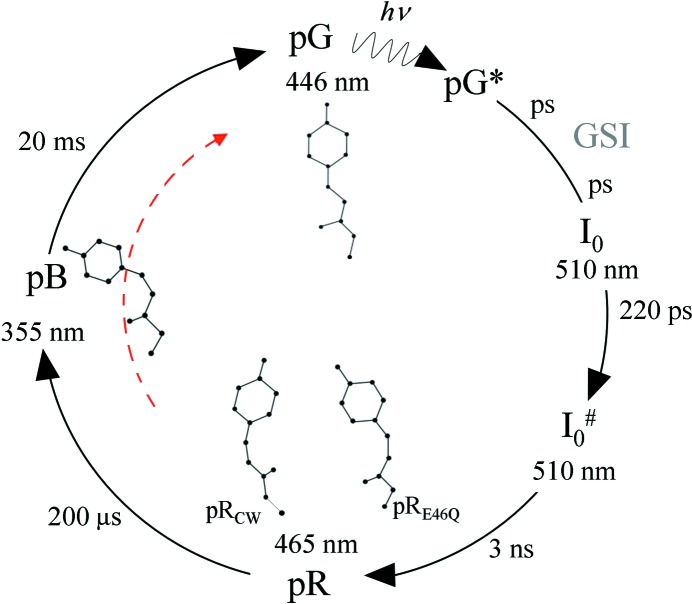
The PYP photocycle. After absorption of a blue photon the photocycle proceeds through a number of intermediates that are occupied on different time-scales. Red dashed arrow: part of the photocycle covered by our short time-series consisting of eight time delays from 256 µs to 32 ms plus the dark data.

Time-resolved macromolecular crystallography (Moffat, 1989 ▶) is a unique method that is able to determine atomic structure and chemical kinetics at the same time (Schmidt, 2008 ▶). X-rays can potentially affect the protein active sites (Dubnovitsky *et al.*, 2005 ▶; Adam *et al.*, 2004 ▶; Purwar *et al.*, 2011 ▶; Schlichting *et al.*, 2000 ▶). Techniques like cryo-cooling (Kuzay *et al.*, 2001 ▶; Nicholson *et al.*, 2001 ▶) and free-radical scavengers (Murray & Garman, 2002 ▶) are used in macromolecular crystallography to reduce radiation damage. However, little direct information is available on whether and how the protein kinetics is affected by radiation damage. Synchrotron beamlines, which are specialized in time-resolved crystallography (Graber *et al.*, 2011 ▶), provide very specific experimental capabilities for the collection of time-resolved X-ray data: the beam size is much smaller than the crystal size typically used for time-resolved data collection, reciprocal space is covered at random to avoid orientational preferences, the crystal is translated along its axis after the collection of a diffraction pattern to expose a fresh crystal volume to the X-rays, and the X-ray beam impinges the crystal as close as possible to the surface where the extent of reaction initiation by a laser pulse is at a maximum. In addition, extremely intense and ultra-short pulses of polychromatic narrow-bandwidth (pink) X-ray radiation are employed. All of these experimental details characteristic of time-resolved studies have never been taken into account in the calculations of the absorbed dose. To address the impact of radiation damage on the protein kinetics a reaction needs to be selected that can be readily investigated by time-resolved crystallography. The photocycle of the photoactive yellow protein (PYP; Meyer, 1985 ▶) provides such a reaction. PYP absorbs a blue photon and enters a photocycle with several intermediates spanning timescales from pico­seconds to milliseconds (Fig. 1 ▶). The photocycle is very well investigated by both time-resolved spectroscopy (Hoff *et al.*, 1994 ▶, 1999 ▶; Rubinstenn *et al.*, 1998 ▶; Ujj *et al.*, 1998 ▶; Hendriks *et al.*, 1999 ▶; Brudler *et al.*, 2001 ▶; Xie *et al.*, 2001 ▶; Borucki *et al.*, 2002 ▶; Shimizu *et al.*, 2006 ▶; van Wilderen *et al.*, 2006 ▶) and time-resolved crystallography (Schmidt *et al.*, 2004 ▶; Anderson *et al.*, 2004 ▶; Ihee *et al.*, 2005 ▶; Genick *et al.*, 1997 ▶). The late intermediates are pB1 and pB2 (Ihee *et al.*, 2005 ▶), which are almost identical in structure except at the N-terminal end (Harigai *et al.*, 2003 ▶; Ihee *et al.*, 2005 ▶; Ramachandran *et al.*, 2011 ▶). The majority of PYP molecules in the crystal revert directly to the dark state (pG) from the pB1 state. The relaxation time from pB1 to pG can be observed exquisitely well with time-resolved crystallographic methods. The idea here is to collect a short time-series consisting of a very limited number of time points on the slower part of the PYP photocycle (see red dashed arrow in Fig. 1 ▶) so that the absorbed dose can be regarded as roughly constant through the short time-series. This time-series is subsequently recollected multiple times until the diffraction patterns fade away. The kinetic analysis on each short time-series will be based on the singular value decomposition (SVD) of time-resolved X-ray data (Schmidt *et al.*, 2003 ▶) and kinetic refinement will be attempted by ‘posterior analysis’ (Schmidt *et al.*, 2004 ▶; Schmidt, Ihee *et al.*, 2005 ▶; Schmidt, 2008 ▶).

## 2. Material and methods   

### 2.1. Time-resolved experiments   

**Figure 2:**
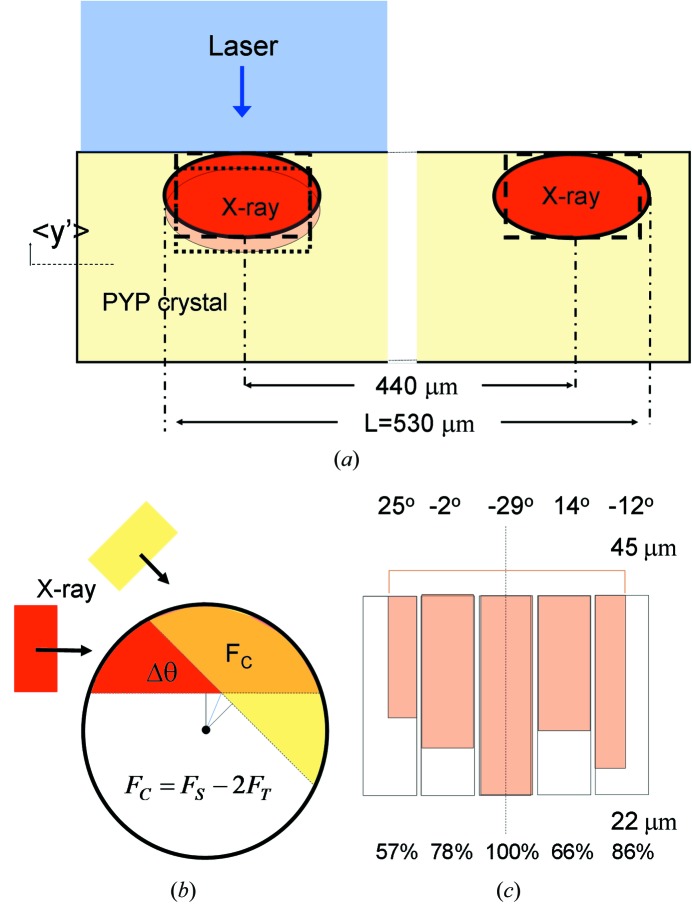
(*a*) Geometry of the crystal setting and X-ray and laser illumination. The X-ray beam (red ellipse) probes the volume near the surface of the crystal that is illuminated by the laser light. The laser beam (blue) is substantially larger than the X-ray beam to facilitate alignment. Arrow: positive *y*′-displacement of the crystal. The region shaded orange shows the new beam position after *y*′-displacement. The crystal translation along the long axis is 440 µm. The length *L* is used for the dose calculation. Dashed box: approximation of the beam with a rectangular box. Dotted box: displacement of the box to the new beam position. (*b*) Model used to determine the common area *F*_C_ used to calculate the common volume *V*_C_. A rectangular X-ray beam (red) whose vertical size is smaller than half the crystal diameter falls on a crystal with a circular cross section. As the crystal is re-oriented by Δθ it is irradiated from another direction (yellow). The orange area is the common area *F*_C_ that determines the common volume *V*_C_. *F*_S_ is the first term and 2*F*_T_ the second term in equation (1), respectively (see also the supplementary material). (*c*) A sequence of angular settings (in degrees). The angular settings are also separated by translations of 22 µm. Five settings are fully or partially exposed given the horizontal size of the X-ray beam. The orange bars denote the relative sizes of the common volumes *V*_C_, for each angular setting, values of which are given as a percentage at the bottom.

Crystals of PYP were grown as described elsewhere (Borgstahl *et al.*, 1995 ▶). A pencil-shaped crystal of dimensions 170 µm × 170 µm × 700 µm was equilibrated in stabilization buffer at pH 7 and mounted in a capillary, with the long dimension along the length of the capillary. This crystal was used in a time-resolved crystallographic experiment using the Laue method at BioCARS beamline 14-ID-B (Graber *et al.*, 2011 ▶) at the Advanced Photon Source, Argonne National Laboratory, USA. The crystal was kept at 288 K using an Oxford Instruments CryoJet. A reaction in the crystal was initiated using a 4 ns (FWHM) laser pulse from an Opolette HEII laser synchronized to the 100 ps X-ray pulses. The pulse energy density of the laser at the crystal was 5 mJ mm^−2^, with the laser beam focused into a 205 µm spot perpendicular to the X-ray beam [see Fig. 2 ▶ and also Fig. 3 in Schmidt *et al.* (2010 ▶) for the crystal–laser X-ray geometry]. Single 100 ps X-ray pulses were extracted by an ultrafast X-ray chopper (Graber *et al.*, 2011 ▶) in the hybrid mode of operation of the synchrotron storage ring. One single pulse contained 3.2 × 10^10^ polychromatic photons with an average wavelength of 1.05 Å, which corresponds to an average photon energy of 11.8 keV. The bandwidth was 10%. The X-ray beam size at the sample was 90 µm (horizontal) × 60 µm (vertical). The scattered radiation from *N*_p_ = 4 X-ray pulses was accumulated on a Mar165 CCD detector to constitute a diffraction pattern. The laser was fired at a time delay Δ*t* before each X-ray pulse. The delay time was varied from 256 µs to 32 ms on a logarithmic timescale. Consequently, a short time-series consisted of *N*_t_ = 8 time points (delay times of 256 µs, 512 µs, 1 ms, 2 ms, 4 ms, 8 ms, 16 ms and 32 ms) plus one Laue pattern where the laser was not fired (dark data). After a time-series was collected from one crystal setting, the crystal orientation was changed to cover more of the reciprocal space. Twenty different crystal orientations (*N*_set_) were used for a dataset. For each new crystal orientation the crystal was also translated along its long axis to expose a fresh volume. For the 20 orientations the crystal was translated a total of 440 µm. Hence, crystal settings were separated by 440 µm/*N*_set_ = 22 µm. In order to compare the protein kinetics and assess the effect of radiation damage, this experiment was repeated 12 times.

As a control, a crystal of size 150 µm × 150 µm × 900 µm was illuminated by laser pulses only with the same pulse energy as above. The protocol used was essentially the same as above, which includes 20 crystal orientations, the same crystal translations (440 µm) and four laser pulses per setting with 5 mJ mm^−2^ per pulse into a spot of about 200 µm, except that we did not expose the crystals to X-rays after the laser illumination. A total of 128 virtual time-resolved datasets were collected which corresponds to 16 virtual time-series, with the crystal exposed only to laser pulses. The progress of the laser damage was monitored by several Laue datasets that were collected exposing the crystals to X-rays in the dark. After an initial dark dataset was collected, we collected four more dark datasets in intervals of four virtual time-series equivalent to 32 virtual datasets or 128 laser pulses per crystal setting. At the end of the experiment the crystal was exposed to a total number of 10240 laser pulses, with 512 laser pulses per crystal setting and five dark Laue datasets collected at equal time intervals.

### 2.2. Average absorbed dose calculation   

To calculate the absorbed dose in J kg^−1^ = Gy we used the program *Raddose* (Murray *et al.*, 2004 ▶). If a crystal of PYP (hexagonal *P*6_3_, *a* = 66.9 Å, *b* = 66.9 Å, *c* = 40.8 Å, six molecules in the unit cell) is irradiated by a 90 µm (h) × 60 µm (v) (full width at half-maximum, FWHM) single X-ray pulse containing 3.2 × 10^10^ photons with an average wavelength of 1.05 Å the absorbed dose is 0.244 × 10^4^ Gy for the X-ray-illuminated voxel equal to the beam size times the thickness of the crystal. The total irradiated crystal volume in the experiment is shown in Fig. 2(*a*) ▶. It is given by the vertical beam size, the thickness of the crystal and the length *L*. *L* is given by the total translation of the beam plus half the beam size at the start and end positions each. In our case, *L* is 530 µm. Hence, each single X-ray pulse adds 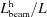 × 0.244 × 10^4^ Gy = 0.0414 × 10^4^ Gy to the total irradiated volume, where 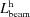 is the horizontal beam size of 90 µm. Each short time-series consisting of *N*_t_ = 8 time delays, the dark data and the edge scan (see below) was collected with *N*_p_ = 4 pulses per crystal setting for a total of *N*_set_ = 20 crystal settings spanning the total translation of the crystal. An average of *N*_P_ *N*_set_(*N*_t_ + 2) × 0.0414 × 10^4^ Gy = 3.3 × 10^5^ Gy would be absorbed per short time-series by the total irradiated crystal volume. Since the experiment was repeated 12 times, complete datasets for 12 short time-series consisting of eight time points plus the dark data were collected. This amounts to 108 complete Laue datasets collected from the same crystal. The total absorbed dose is therefore 12 × 3.3 × 10^5^ Gy = 39.6 × 10^5^ Gy or about 4 MGy. Since one X-ray pulse lasts only 100 ps, the instantaneous (pulse) dose rate *R*_inst_ is 0.244 × 10^4^ Gy/(100 × 10^−12^ s) = 2.4 × 10^13^ Gy s^−1^. The average dose rate *R*_ave_ is 0.244 × 10^4^ Gy/4 s = 610 Gy s^−1^ because the waiting time between the single pulses was 4 s, and the time delays are negligible in comparison.

### 2.3. Corrections to the dose absorbed   

Corrections to the average dose calculated above are necessary as explained in this section. As they are due to additional fresh crystal volume being exposed to X-rays, they effectively reduce the average absorbed dose calculated above (Table 1 ▶).

#### 2.3.1. Crystal rotations   

Our goal was to find out how many datasets can be safely collected on a PYP crystal exploiting the capabilities of the BioCARS 14-ID beamline and data collection protocol. A very useful feature is the random coverage of reciprocal space. The idea is that subsequent settings of the crystal are maximally spaced across reciprocal space until a complete dataset is collected. If the experiment terminates prematurely, the data have no preferred orientation and, although incomplete, might already provide meaningful difference electron density maps. A consequence of this feature is that subsequent crystal orientations are largely different. As we rotate the crystal and the crystal is thick compared with the vertical X-ray beam size, some fresh crystal volume is introduced. We used a relatively simple model to correct for this. In Fig. 2(*b*) ▶ a circle is shown representing a cross section through the crystal. We impinge this crystal with an X-ray beam [red in Fig. 2(*b*) ▶] whose vertical size *v* is smaller than the radius *t*/2 of the crystal. When the crystal is rotated by an angle Δθ, another volume is illuminated by the yellow beam. The red and the yellow beams share a common area *F*_C_ (orange) which depends on the angular difference Δθ of the beam directions, and which can be calculated as 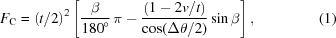
with β = 90° − Δθ/2 − arcsin(1 − 2*v*/*t*). Equation (1) and the equation for the relative common volume (in %) are derived in the supplementary material.^1^

Fig. 2(*c*) ▶ shows an actual sequence of crystal settings used in our experiments, each of which is separated by an angle θ, the above-mentioned translation of 22 µm. The X-ray beam is shown for the central setting at −29°. Since the horizontal beam diameter is 90 µm, the beam covers three full settings plus the two flanking one only partially. So, five settings are affected. All five neighboring settings share common volumes depending on their angular settings. The correction factor for the dose absorbed by an entire small time-series is the average over all sets of five-membered neighboring common volumes.

#### 2.3.2. Edge scan   

A convenient feature of the data collection protocol is the edge scan. Here, the crystal is translated along the vertical direction (across the X-ray beam) and one single X-ray pulse is used to produce a weak diffraction pattern. Once the X-ray beam crosses the edge of the crystal, the diffraction patterns fade away. The analysis of such a series of diffraction patterns collected across the crystal edge is based on the totally scattered intensity in each pattern. It can determine the position of the edge precisely. The crystal is then positioned in the X-ray beam such that only the surface layer of the crystal is probed by the X-rays (Schotte *et al.*, 2003 ▶). Fig. 2(*a*) ▶ shows the geometry. This is necessary, since protein crystals are exquisitely optically thick and the laser light penetration into the crystal is therefore shallow. As a result, the laser pulses initiate the reaction primarily close to the illuminated crystal surface. The edge scan helps to maximize the overlap of the X-ray beam with the laser-illuminated volume. The edge scan was performed once, at the beginning of each crystal setting, hence 20 times per short time-series. The position *x*,*y* of the goniometer is recorded after each edge scan. These coordinates were used together with the angular setting θ of the goniometer to calculate the relative vertical displacements *y*′ of the crystal across the X-ray beam for each crystal setting. The individual *y*′ were averaged (〈*y*′〉, see Table 1 ▶). The crystal displacement about 〈*y*′〉 exposes a new crystal volume to the beam. The dose calculation needs to be corrected. For simplicity, we assumed that the correction owing to edge scan and that owing to crystal rotation can be applied independently, and that their effects add. For the correction, we assumed a rectangular X-ray beam shape with the same area and the same vertical size as the ellipsoidal realistic X-ray beam (Fig. 2*a* ▶). A displacement of 10 µm exposes 17% of fresh volume and 83% is exposed to the dose from the previous sweep(s). Using 〈*y*′〉 we can correct for the dose, subsequently for each sweep. The dose necessary for the edge scan itself is roughly equal to that of one regular diffraction pattern (about four single X-ray shots were used within the crystal volume) and this was already taken into account in the average absorbed dose calculation.

We also include the edge scan in our control experiment where we exposed the crystal to the laser pulses only. The edge scan was performed for the five dark datasets collected at the mentioned regular time intervals. The crystal positions from those edge scans are stored and applied for the virtual time-series with laser exposures only. In this way we assessed whether subsequent laser damage results in displacements of the crystal deeper into the X-ray beam.

### 2.4. Data reduction and data analysis   

Laue data were indexed and integrated using the program *Precognition* and scaled using *Epinorm* (both RenzReserach, http://renzresearch.com/). Difference structure factor amplitudes were calculated as reported (Ren *et al.*, 2001 ▶; Ihee *et al.*, 2005 ▶). Protein Data Bank (Berman *et al.*, 2002 ▶) entry 2phy was used to provide phases to calculate time-dependent difference maps. Twelve short time-series were obtained, each subject to a higher absorbed dose (see Table 1 ▶). Each of these time-series was analyzed by SVD (Schmidt *et al.*, 2003 ▶; Zhao & Schmidt, 2009 ▶). For this analysis only the difference electron density in the chromophore region was included. Grid points that include the chromophore and the amino acid residues that line the chromophore pocket such as Tyr-42, Glu-46, Met-100 and Arg-52 (in total 188 atoms) were masked out. The mask was modified by allowing only grid points that are above 2.5σ or below −2.5σ in at least one of the difference maps of the small time-series. The masked difference maps were arranged into data matrix *A*, which was decomposed by SVD into the left (*U*) and right (*V*) singular vectors and the corresponding singular values (*S*) according to *A* = *USV* ^*T*^. The kinetics of the reaction is observed in the right singular vectors (RSVs). The first RSV for each short time-series was fitted by a sum of two exponentials, one exponential for a rising phase (if present) and another for a decaying phase. The characteristic time τ_RSV_ for the decaying phase is reported.

### 2.5. Posterior analysis   

**Figure 3:**
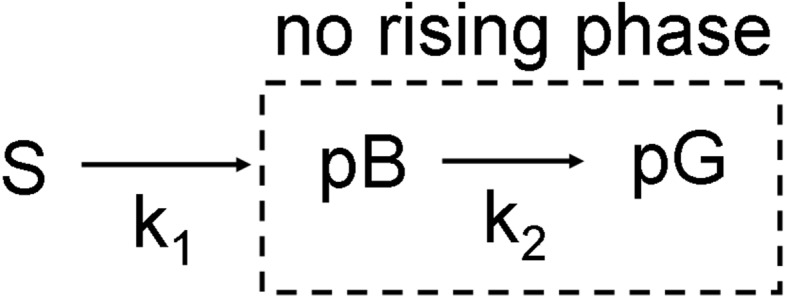
Simple kinetic mechanism with a source *S* and two rate coefficients *k*_1_ and *k*_2_ for kinetic refinement with posterior analysis. Dashed box: mechanism used if no rising phase is detected in the RSV.

Posterior analysis refines the rate coefficients for a given mechanism by comparing calculated difference maps that depend on the rate coefficients of the mechanism with the observed difference maps. In the case of this study the intermediate that is dominant throughout the time-series is pB1. There is also some small pR remnant at the beginning of our time-series (256 µs), but the majority of the electron density features must be interpretable by only pB1. Since the pB1 structure is known (Ihee *et al.*, 2005 ▶; Tripathi *et al.*, 2012 ▶), we can calculate the time-independent pB1 − pG difference map 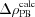. If a raising phase was observed in the first RSV (see below), we calculated the time-dependent fractional concentration c_frac_(*k*,*t*) based on a mechanism involving a rate coefficient *k*_1_ for the pB1 state formation from the source state *S* and a rate coefficient *k*_2_ for a subsequent pB1 state decay (see Fig. 3 ▶). The time-dependent difference maps are then calculated as
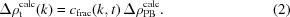
By fitting these maps to the observed difference maps [equation (3)], the concentration profile of pB1 is reflected properly even without knowing the structure of the source *S*,
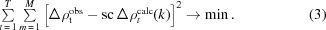
Here the fit is executed at *M* grid points in a mask similar to the one used to perform the SVD, and *T* = 8 representing time points from 256 µs to 32 ms. The scale factor sc represents the peak fractional concentration of molecules in the pB1 state and is, as well as *k*_1_ and *k*_2_, a fit parameter. If the rising phase was not observed in the first RSV, we used a mechanism where pB1 only decays with *k*_2_ (dashed box in Fig. 3 ▶). In all cases the magnitude of *k*_2_ is compared depending on the absorbed X-ray dose. Posterior analysis was performed using the program *GetMech* (Schmidt *et al.*, 2004 ▶; Schmidt, 2008 ▶).

## 3. Results   

The mean intensity 〈*I*〉 is the generally used metric to address radiation damage (Owen *et al.*, 2006 ▶; Southworth-Davies *et al.*, 2007 ▶). Our data were processed up to 1.6 Å. However, since data quality is poor in the last resolution shell, we used data to 1.8 Å to calculate the difference maps. In order to address the radiation damage we calculated 〈*I*〉 as well as the mean of the ratio of the intensity over its experimental uncertainty 〈*I*/σ_*I*_〉 in a resolution shell from 1.9 to 1.8 Å. We used unscaled raw reflection intensities derived from integrating the Laue spots of the diffraction pattern. In this way we avoided any scaling and merging and a clean estimate of 〈*I*〉 as well as 〈*I*/σ_*I*_〉 of the Laue data was obtained. The uncorrected dose increased in steps of 3.3 × 10^5^ Gy per time-series up to about 4 MGy in our final time-series.

The absorbed dose was adjusted based on an estimate of the extent of fresh crystal volume exposed owing to subsequent crystal settings. 77% of the total volume is shared (*V*_C_ = 77%), hence the different crystal orientations (settings) exposed 18% of fresh volume on the average in addition to the translation. The dose was further adjusted based on our recorded vertical displacements of the crystal (see Fig. 2 ▶ and Table 1 ▶). For the first five time-series the displacements are negligible, then the crystal started to move up. At time-series 12 (the last sweep), 〈*y*′〉 was 16 µm relative to the position of time-series 1; hence, from time-series 5 to 12 a total of 25% of new volume is eventually exposed (see Table 1 ▶ for the corrected dose).

**Figure 4:**
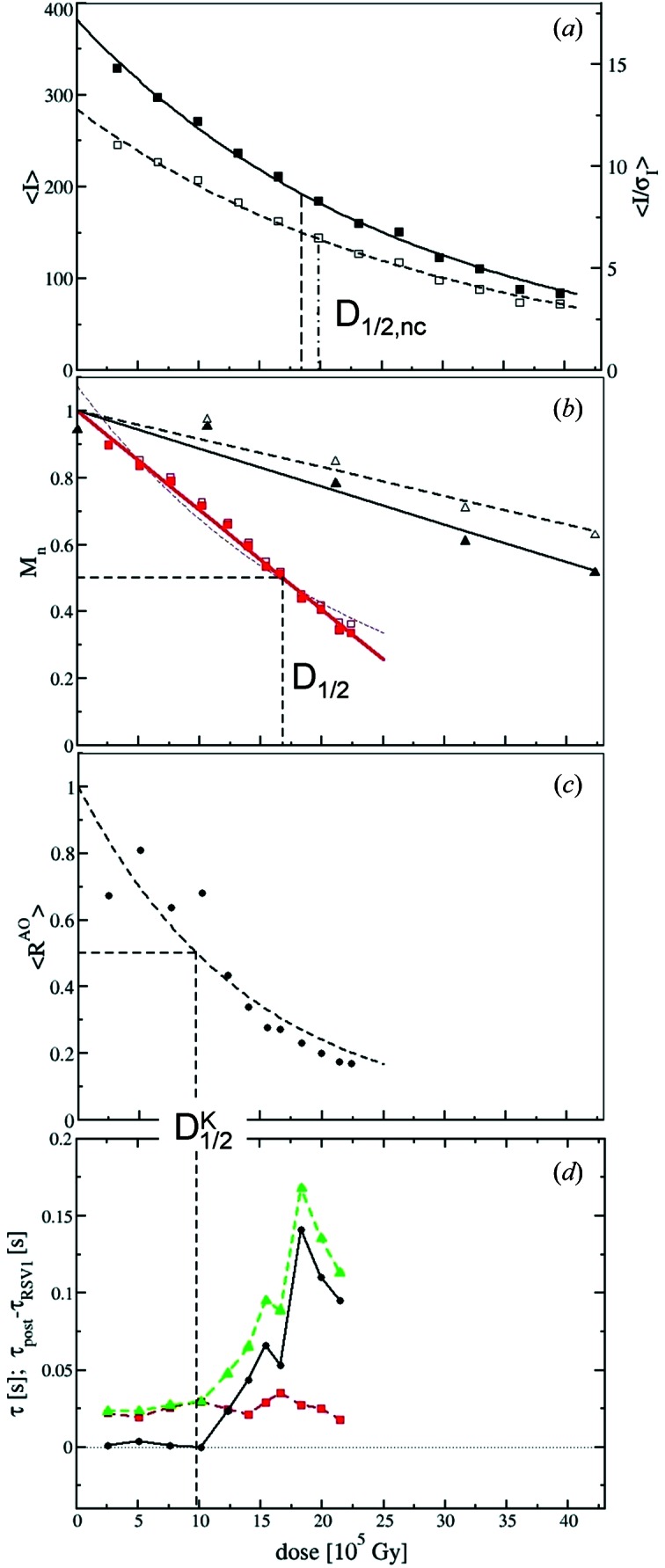
(*a*) Raw mean intensities 〈*I*〉 (solid squares) and 〈*I*/σ_*I*_〉 (open squares) as a function of the uncorrected dose. Black and dashed lines: fits by exponential functions. Vertical dashed and dashed-dotted lines indicate the *D*_1/2_ obtained with the uncorrected dose. (*b*) Normalized quantities *M*_n_ plotted as a function of adjusted dose. Average intensity 〈*I*〉_free_ (red solid squares) and 〈*I*/σ_*I*_〉_free_ (red open squares) are plotted as a function of adjusted dose. Red solid line: fit by a straight line. Thin dotted line: fit by an exponential function. The dashed vertical line indicates the corresponding *D*_1/2_. Black solid and black open triangles: variation of the mean intensity 〈*I*〉_L_ and 〈*I*/σ_*I*_〉_L_, respectively, observed in the control experiment as a function of virtual dose. The black solid and dashed lines are fits by a straight line. (*c*) Normalized *R* ^AO^ values (black spheres) as a function of adjusted dose. Black dashed curve: fit by a single exponential. The horizontal/vertical dashed lines indicate 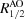 and the corresponding kinetic dose limit 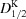. (*d*) Red squares: relaxation times τ_RSV1_ from the SVD analysis as a function of adjusted dose. The red dashed line is a guide to the eye. Green triangles: inverse of the rate coefficient (relaxation time τ_post_) obtained from posterior analysis. The green dashed line is a guide to the eye. Black spheres: difference between τ_post_ and τ_RSV1_. The dashed vertical line indicates the kinetic dose limit 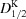.

In our control experiment (laser pulses only) we observed a linear decay of the 〈*I*〉 as well as of the 〈*I*/σ_*I*_〉 values as a function of the laser pulses. In Fig. 4(*b*) ▶ we have translated the number of laser pulses into virtual X-ray dose, which is the dose absorbed by the same crystal if it were exposed to X-rays. This way we can plot 〈*I*/σ_*I*_〉 (or 〈*I*〉) observed in our control experiment in the same frame as the average intensities of the true time-series [see solid triangles in Fig. 4(*b*) ▶]. We corrected the virtual dose by a common volume calculation similar to that for the real data, only here we used a smaller crystal with *t* = 150 µm. The common volume is slightly larger (80% compared with 77% in the real time-series), and the corresponding virtual dose is also slightly larger. There was no need to further correct for crystal displacements into the X-ray beam. In fact, the crystal position remained surprisingly stable until the final dark X-ray exposure. The 〈*I*〉 values (solid black triangles) were fit by a straight line with slope *S*_L_ = −0.0113 × 10^−5^ Gy^−1^ which is normalized to unity at zero virtual dose. This line reaches half of its initial value at the virtual value half value dose 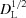 of about 4.5 MGy. In the dose range of our real data [red solid squares in Fig. 4(*b*) ▶] the intensity decay owing to laser damage is small but meaningful as it reaches 20% at ∼2.0 MGy. Assuming that laser damage and X-ray damage are independent events, we can correct the intensity of our true time-series for the laser damage and obtain intensities that are free of laser damage: 〈*I*〉_free_ = 〈*I*〉(−*S*_L_ *D*_corr_ + 1), where *D*_corr_ is the dose of our time-series corrected by the common volume and the edge scan and *S*_L_ is given above. Since *S*_L_ is negative, the 〈*I*〉_free_ values are slightly higher than those determined from the raw intensities of our time-series (see Table 1 ▶).

In Fig. 4(*a*) ▶ (red squares) the 〈*I*〉 and 〈*I*/σ_*I*_〉 values from datasets collected at the 32 ms time-delay from 12 repeated time-series are shown as a function of the uncorrected dose (solid and open squares, respectively). The data are fitted by a single exponential [solid lines in Fig. 4(*a*) ▶]. The half-value uncorrected dose *D*_1/2,nc_ is obtained when this exponential decays to half of its initial value, which is after 18.4 × 10^5^ Gy when the 〈*I*〉 values and 19.7 × 10^5^ Gy when the 〈*I*/σ_*I*_〉 values are used (see Table 2 ▶). In Fig. 4(*b*) ▶ the dose has been corrected. The laser-damage-free 〈*I*〉_free_ (solid red squares) and the 〈*I*/σ_*I*_〉_free_ from Table 1 ▶ (open red squares) were normalized to unity at zero dose and the values are plotted as a function of the corrected dose. In the dose interval assessed by our experiments the decay follows more a straight line [see the fit of an exponential which is shown by the thin dashed line in Fig. 4(*b*) ▶]. Also here, the half value dose *D*_1/2_ can be obtained when the data decay to half their initial magnitude. *D*_1/2_ is the same, 16.7 × 10^5^ Gy, regardless of whether 〈*I*〉 or 〈*I*/σ_*I*_〉 values are used (Table 2 ▶).

**Figure 5:**
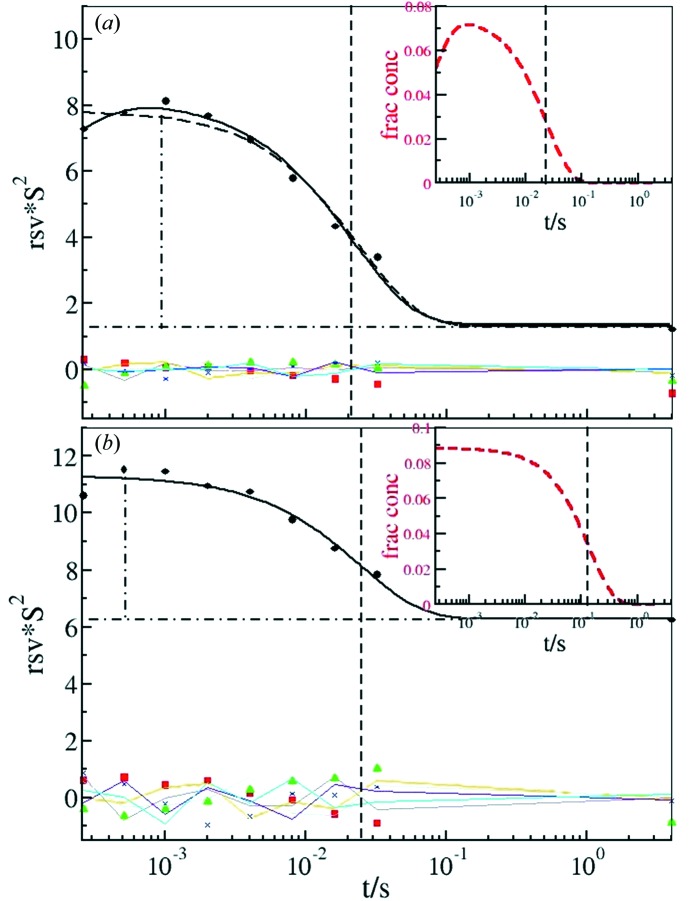
Right singular vectors (RSV) resulting from the SVD analysis of the short time-series. All singular vectors are shown. (*a*) First short time-series with the lowest absorbed dose. Solid spheres: first singular vector; solid squares: second singular vector; solid triangles: third singular vector; blue crosses: fourth singular vector. RSV 5 to 8 are shown as thin lines. Solid black line: fit of two exponentials with a source and decaying phase. Vertical dashed line: relaxation time of the decaying phase from a fit of the sum of two exponentials; long dashed line: fit of only one exponential with the same relaxation time; vertical dashed-dotted line: amplitude of RSV1; horizontal dashed-dotted line: offset of RSV1. Insert: red dashed curve: concentration profile of pB1; black dashed line: relaxation time from the inverse of the rate coefficient *k*_2_. (*b*) Tenth short time-series with a high absorbed dose. Solid spheres: first singular vector; solid squares: second singular vector; solid triangles: third singular vector; blue crosses: fourth singular vector. RSV 5 to 8 are shown as thin lines. Vertical dashed line: relaxation time of the decaying phase from fit of only one exponential; vertical dashed-dotted line: amplitude of RSV1; horizontal dashed-dotted line: offset of RSV1. Insert: red dashed curve: concentration profile of pB1; black dashed line: relaxation time from the inverse of the rate coefficient *k*_2_.

**Figure 6:**
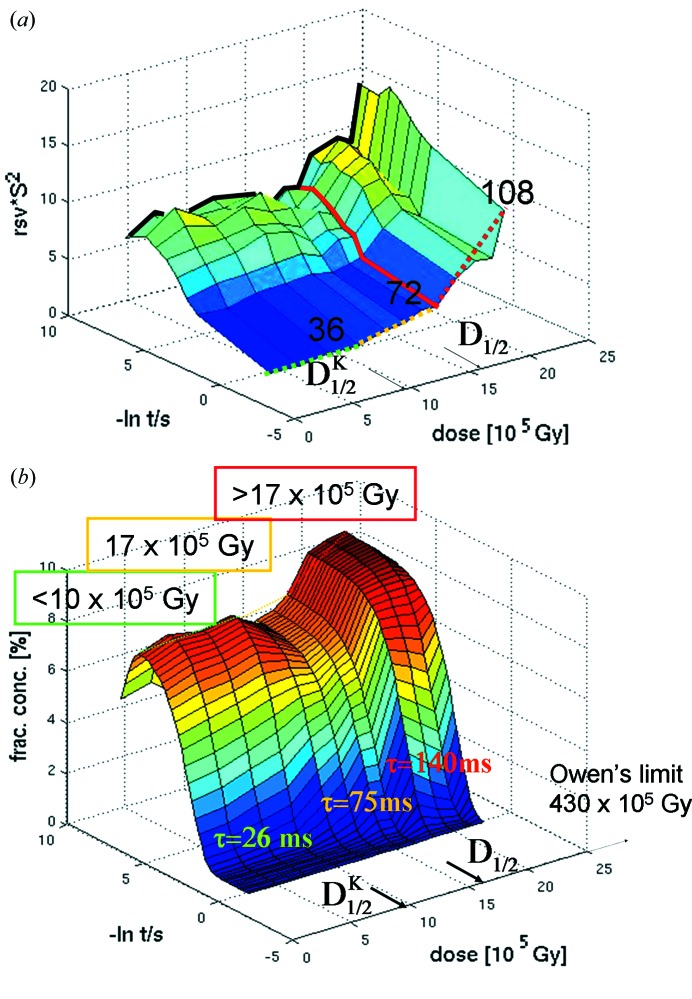
Three-dimensional plot of first right singular vectors for all 12 short time-series shown as a function of dose. 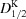 is reached after 36 datasets or four short time-series. The offsets in the RVS are indicated by the dotted lines. Green dotted line: small offsets; orange dotted line: offset increases slightly; red dotted line: offset increases strongly. The orange regime ends after 72 datasets. The red line indicates that posterior analysis of the data beyond this dose will not be possible. *D*_1/2_ is also indicated. (*b*) Three-dimensional plot of all fitted time courses from the posterior analysis as a function of dose. The green, orange and red regimes as well as the approximate relaxation times that can be expected in these regimes are marked. 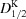, *D*_1/2_ as well as Owen’s limit are also shown.

However, it is unknown whether the *D*_1/2_ value is also a meaningful limit for the PYP photocycle kinetics. To address this we subjected the light–dark difference maps to the SVD. Fig. 5 ▶ shows the RSVs for the first time-series, with an average absorbed dose of 2.5 × 10^5^ Gy, compared with those of the tenth time-series with 20 × 10^5^ Gy. Since our time-series span the slow part of the PYP photocycle and only pB1 is dominant, only one significant RSV (RSV1) is present in each small time-series. The difference between RSV1(1) and RSV1(10) is immediately evident. For both time courses the first singular vectors were fit by trial functions, which were independently determined based on the appearance of the first RSV. For time course 1 two exponentials were employed, one with a rising phase and another displaying a decay with a relaxation time τ(1)_RSV_ (Fig. 5*a* ▶). For comparison, a single exponential is also shown in Fig. 5(*a*) ▶ (long dashed lines) with the same τ(1)_RSV_. In time course 10 a rising phase can barely be observed in the first RSV1(10) and a fit of an exponential to this rising phase was not possible. Consequently, only one exponential with the characteristic time τ(10)_RSV_ was used. Interestingly, both τ(1)_RSV_ and τ(10)_RSV_ are very similar, although the amplitudes and offset of RSV1(1) and RSV1(10) differ grossly [compare Figs. 5(*a*) and 5(*b*) ▶]. Typically, with increasing radiation damage there is an increasingly higher level of overall difference electron density in the maps, which is accumulated in the first left singular vector. This can also be observed in the offset of the corresponding first RSV and is also a reported effect in spectroscopy for data with reduced signal-to-noise ratio (Henry & Hofrichter, 1992 ▶). This offset is large in RSV1(10) and small in RSV1(1) (Fig. 5 ▶). Consequently, the ratio, *R* ^AO^, of the amplitude of the first RSV to the offset is large for RSV1(1) and much smaller for RSV1(10). For RSV1(1), *R* ^AO^ is 6 and, for RSV1(10), *R* ^AO^ is only 1.5. In Fig. 6(*a*) ▶ the first RSVs for all the time courses are shown in a three-dimensional plot. The corresponding *R* ^AO^ are shown in Fig. 4(*c*) ▶ and listed in Table 1 ▶. The decaying *R* ^AO^ can be fit by a single exponential [black dashed curve in Fig. 4(*c*) ▶]. From the fit, 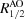 is determined. Only the first four time-series have *R* ^AO^ values larger than 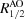. We call the corresponding dose (9.7 × 10^5^ Gy) the kinetic half-value dose (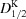; see also Table 2 ▶).

*R* ^AO^ is a good indicator of the outcome of the post-SVD refinement of the mechanism. Up to 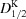, the relaxation times τ_SVD_ found in the RSV(1) [Fig. 4(*d*) ▶, red squares] agree with relaxation times τ_post_ which are the inverse of the rate coefficients *k*_2_ from the posterior analysis [green triangles in Fig. 4(*d*) ▶]. After 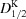, τ_RSV_ and τ_post_ diverge. Hence, although the timescale of the reaction is found to be 25 ms by fitting the RSV1, posterior analysis will shift this timescale to more than 150 ms if the absorbed dose is too high [Table 1 ▶ and Fig. 6(*b*) ▶]. On the other hand, at low to moderate doses up to 9.7 × 10^5^ Gy (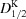), time-scales from the SVD and posterior analysis agree very well [Fig. 4(*d*) ▶, black spheres].

## 4. Discussions   

We inspected the difference map calculated from the dark data at time-series 1 and 12 (*D*1 − *D*12 difference maps) for site-specific radiation damage of the dark PYP (Fig. S2, supplementary material). However, we were unable to identify damage on any amino acid residues of the PYP (including Asp, Glu or the sulfur-bearing amino acids). The only effect is that the difference maps become noisier. The noise level in the *D*1 − *D*2 difference map is 0.012 e^−^ Å^−3^ which in the *D*1 − *D*12 difference map is higher by a factor of three (0.032 e^−^ Å^−3^). We also do not observe any specific damage when the difference map *D*5 − *D*1 from our control experiment (laser only) is inspected (see also Fig. S2, supplementary material). It appears that in this case all damage is non-specific. Probably, unlike at cryogenic temperatures where the molecules are stabilized, at room temperature the PYP molecules quickly lose their structural integrity when damaged. Even if there is specific radiation damage, its signature disappears as if the entire molecule is removed from the crystal resulting in crystal imperfection and increasingly larger *B*-factors (Rajendran *et al.*, 2011 ▶). This is further corroborated by the observation that the PYP Laue reflection patterns become more streaky with higher X-ray doses. Streaks in the Laue pattern result from enlarged crystal mosaicity. A possible reason for this is that damaged PYP molecules create defects in the crystals which in turn generate long-range disorder and increased mosaicity. Interestingly, others observe specific damage also at room temperature (Kmetko *et al.*, 2011 ▶) even at moderately high doses around 1 × 10^5^ Gy which is one order of magnitude lower than our *D*_1/2_. Damage is most prominent on disulfide bonds, of which PYP has none, and there are, as in our difference maps, noise features scattered throughout.

When monochromatic data are processed, the number of reflections found by the software typically decreases with decreasing scattering power (Owen *et al.*, 2006 ▶; Rajendran *et al.*, 2011 ▶). This discourages the use of 〈*I*/σ_*I*_〉 values and favors the use of mean intensities to study dose effects. In Laue crystallography, however, the data reduction software determines the number of reflections based on the form of the wavelength normalization curve (Ren & Moffat, 1995 ▶). The number of predicted reflections stays approximately the same even if the dose is increased and the scattering power of the crystal decreases (see Table 1 ▶). As a consequence, the *D*_1/2_ determined with the 〈*I*〉 or the 〈*I*/σ_*I*_〉 are essentially the same. With Laue data the 〈*I*/σ_*I*_〉 can be used instead of, or in addition to, the mean intensities.

Since the crystal was not rotated during X-ray exposure, the dose calculated by *Raddose* for each exposure is accurate (Garman & Weik, 2011 ▶). However, the average absorbed dose, based on the simple initial calculation that took into account crystal translation during collection of each short time-series (see §2), was adjusted twice to account for effects caused by the additional introduction of fresh crystal volume during the data collection. The first contribution comes from the fresh crystal volume that is exposed each time the crystal orientation, the angular setting, is changed. Maximizing this volume will decrease the dose per dataset and will allow more datasets to be collected. Taking crystal symmetry and space-group considerations into account, subsequent crystal orientations need to be as far apart as possible to make use of the entire available crystal volume. The second contribution to the dose adjustment comes from the vertical translation of the crystal relative to the X-ray beam when the dose increases. The edge scan, which is used to position the crystal in the X-ray beam, is based on the total scattered intensity which in turn is affected by the dose. The result is that the X-ray beam moves deeper into the crystal, away from the crystal surface. This might pose a problem for time-resolved experiments, since the X-ray beam increasingly probes deeper regions of the crystal that are not optimally illuminated by the laser beam. However, below 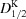, the crystal displacements remain negligible, smaller than 1 µm on average, and the edge scan can be safely used to optimize the overlap between the X-ray beam and the laser-illuminated volume of the crystal.

After the dose adjustments the initial exponential decrease of the intensities (or *I*/σ_*I*_ values) appears to be linear [compare Figs. 4(*a*) and 4(*b*) ▶]. Our linear decay may indicate that the mechanism for damage at room temperature and close to the surface is different from proposed first-order models (see Southworth-Davies *et al.*, 2007 ▶). To fully account for this, more experiments are necessary.

The SVD analysis of time-resolved data on PYP is amazingly robust against radiation damage. The relaxation times extracted from the SVD show almost no dose dependence even after 2 MGy at room temperature (Fig. 4*d* ▶). This is because the SVD deals properly with the increasingly larger difference electron density background (offset) on which a subsequently smaller signal is sitting (see Fig. 5 ▶). Since the SVD is able to accurately deal with very small occupancies of the order of 3–5% (Schmidt *et al.*, 2003 ▶), extraction of accurate relaxation times is still feasible at very high doses, far higher than the dose limit *D*_1/2_. However, to extract and refine a proper kinetic mechanism, posterior analysis is necessary. Its outcome is strongly dose-dependent, since at high doses the calculated noise-free difference maps have to be fitted to the very noisy observed difference maps. At higher doses the posterior analysis attempts to fit the noise and produces rate coefficients that are at least a factor of five lower than those at the lowest doses (Table 1 ▶). So care has to be taken to limit the dose. In Fig. 6(*b*) ▶ results from the posterior analysis are shown as a function of dose. We can distinguish three regimes: the green regime goes up to the 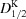, 9.7 × 10^5^ Gy, where the relaxation rates of both SVD and posterior analysis agree. In the orange dose-regime, up to 17 × 10^5^ Gy, which is equal to the *D*_1/2_, some kinetic information can be extracted. Relaxation times between SVD and posterior analysis differ by up to a factor of three. Above that regime (in the red regime), it is questionable whether a complex kinetic analysis is feasible. For PYP the dose should be kept lower than 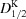 to stay in the green regime of Fig. 6 ▶.

Room-temperature absorbed dose effects were recently reported (Southworth-Davies *et al.*, 2007 ▶; Rajendran *et al.*, 2011 ▶; Kmetko *et al.*, 2011 ▶; Barker *et al.*, 2009 ▶). Results strongly depend on the dose-rate employed to collect the data. We were using an average dose rate of 600 Gy s^−1^ for PYP (see above) and our *D*_1/2_ is 16.7 × 10^5^ Gy which is remarkably similar to the *D*_1/2_ for lysozyme reported by Southworth-Davies *et al.* (2007 ▶) (16.3 × 10^5^ Gy at 10 Gy s^−1^) but much higher than the *D*_1/2_ values reported by Rajendran *et al.* (2011 ▶) on insulin (2.2 × 10^5^ Gy at their lowest dose rate of 1430 Gy s^−1^). The latter study reports a negative effect on the dose rate, hence *D*_1/2_ decreases with increasing dose-rate. Single-pulse Laue experiments use the highest peak dose-rates available (2.3 × 10^13^ Gy s^−1^) at synchrotrons. Obviously, it is not the high peak dose-rate that shows a detrimental effect, but it is the average dose-rate, which was moderate in our experiments (∼600 Gy s^−1^). It was suggested that high dose-rates disproportionately heat up the crystal, which leads to damage. Furthermore, hydrogen is presumably produced, which accumulates in the crystal at higher dose-rates and which may be one of the causes, if not the main cause, for the negative dose-rate dependence (Meents *et al.*, 2010 ▶; Rajendran *et al.*, 2011 ▶). Although our instantaneous dose-rates were extreme, the 4 s waiting time between the X-ray pulses is enough for the crystal to cool down (Moffat *et al.*, 1992 ▶) and for any potential hydrogen to diffuse out of the crystal. This maintains crystal integrity at higher doses.

Our *D*_1/2_ is among the largest reported on a protein crystal at room temperature so far. Still, it is more than an order of magnitude smaller than the Henderson limit (200 × 10^5^ Gy), which, however, is valid only at cryogenic temperatures (Henderson, 1990 ▶). Owen *et al.* (2006 ▶) determined a roughly two times higher cryogenic *D*_1/2_ value of 430 × 10^5^ Gy. It is thought that secondary damage effects such as diffusion of radicals are strongly inhibited at these low temperatures. We achieved here about 1/25 of Owen’s limit although we were operating at room temperature, where free radicals and solvated electrons may diffuse freely. It may well be that with the experimental conditions presented we reached a dose limit for room-temperature X-ray data collection on crystals that are not treated with radical scavengers. As has been shown by others (Barker *et al.*, 2009 ▶), adding radical scavengers such as ascorbate may even increase this limit. A properly set up single-pulsed Laue experiment then becomes a tool to collect diffraction data on a dose-sensitive specimen that also obstinately resists freezing. Experiences with cytochrome-c nitrite reductase crystals, which deteriorate quickly in a monochromatic X-ray beam at ambient temperatures, seem to corroborate this observation. With short polychromatic X-ray pulses it was possible to collect a complete high-resolution dataset at 273 K (Youngblut *et al.*, 2012 ▶). However, to firmly establish this observation, systematic experiments are necessary. One could presume that the dose limit given by short-pulsed Laue crystallography may only be surpassed by diffract-and-destroy experiments at a free-electron laser (Chapman *et al.*, 2011 ▶; Spence & Hawkes, 2008 ▶), where radiation damage occurs after the scattering event and diffraction patterns are essentially damage-free (Chapman *et al.*, 2006 ▶).

Nevertheless, for PYP we determined a kinetic dose limit, 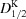. Roughly 36 complete Laue datasets can be collected to 1.6 Å from one moderately sized PYP crystal, the size of which is very well suited to time-resolved experiments, without disturbing the kinetic analysis by post-SVD analysis (see Figs. 4 ▶ and 6 ▶). The rule of thumb is to collect three datasets per order of magnitude in time for a successful SVD analysis (Schmidt *et al.*, 2003 ▶). The 36 datasets within the 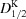 limit, therefore, will allow the coverage of 12 orders of magnitude in time. This requirement can be easily fulfilled when a time-series from 1 ns to 0.5 s is collected on one crystal. This requires 27 time points (datasets) equidistantly spread on a logarithmic timescale plus the dark. There is even some room to increase the number of time points further into the picosecond regime. The experiments shown here demonstrate that entire time-series from picoseconds to seconds can be collected from a single crystal (Schmidt *et al.*, 2010 ▶).

Our results also show that by carefully setting up a time-resolved experiment the number of kinetically meaningful datasets available from a single crystal can be maximized. Although a thicker crystal absorbs more energy for a given incident intensity owing to the longer path of the X-ray beam through the thicker crystal, the dose remains the same since the absorbing volume is also proportionally larger. For intensities scattered into Bragg reflections the situation is different. The intensity scales linearly with mass because the lattice factor is proportional to the number of unit cells. Hence a crystal that is twice as thick scatters with twice the intensity. To match the average intensity scattered by a thin crystal, a thick crystal requires lower incident X-ray beam intensity. Consequently, lower dose is absorbed per diffraction pattern. To reach the dose limit *D*_1/2_ which is the same for a thin and a thick crystal, twice as many diffraction pattern can be collected from a crystal that is twice as thick as a corresponding thin crystal. Since the kinetic dose 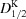 is subject to the same reasoning, we can expect to collect even more kinetically meaningful time-resolved diffraction patterns from a thick PYP crystal up to a reasonable crystal thickness, and the use of thin crystals is discouraged. The thickness is limited by the laser beam diameter along the axis of the X-ray beam. BioCARS 14-ID-B currently features ellipsoidal laser beam profiles with the large axis up to 600 µm (Graber *et al.*, 2011 ▶) along the X-ray beam direction, so a crystal thickness in the range 300–400 µm would be optimal. With this, more than 400/170 × 36 = 85 kinetically meaningful time points can be collected from one PYP crystal. The dose would still only be around 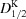. On the other hand, if the number of kinetically meaningful datasets (time points) drops below 27, a comprehensive kinetically meaningful time-series cannot be collected from a single crystal. This would happen when the crystal thickness falls below about 120 µm. In this case, however, longer crystals can be selected where larger lengths *L*′ are possible, and the number of useful datasets increases by *L*′/*L*. These results pave the way to five-dimensional crystallography (Schmidt *et al.*, 2010 ▶), where in addition to space and time an additional parameter such as the temperature or the pH (Tripathi *et al.*, 2012 ▶) is varied. Here, success depends critically on the ability to collect an entire kinetically meaningful time-series from only one crystal.

For PYP the values of 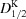 and *D*_1/2_ differ, with 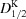 of the order of 40% smaller (Table 2 ▶). This relationship might also hold for proteins other than PYP, which were investigated by a long and comprehensive time-series of time-resolved crystallographic data such as myoglobin (Srajer *et al.*, 2001 ▶; Schmidt, Nienhaus *et al.*, 2005 ▶) or clam hemoglobin (Knapp *et al.*, 2006 ▶). Since *D*_1/2_ can be relatively easily determined beforehand, the number of kinetically useful datasets can then be estimated from the size of the crystal mounted for the time-resolved crystallographic experiment.

## Supplementary Material

Supporting information file. DOI: 10.1107/S090904951105549X/rx5003sup1.pdf

## Figures and Tables

**Table 1:** Absorbed dose (in 10^5^Gy) and data statistics for 12 consecutive short time-series, each consisting of the same eight time delays and a dark data set *y*: average vertical translation of the crystal. The average *I* and *I*/_*I*_ in the resolution shell 1.91.8 were determined from the 32ms time point of each short time-series. *N* is the number of reflections in this resolution shell. For each short time-series, characteristic times for the decay phase are given from the fit to the RSV (_RSV_) and from posterior analysis (1/*k*_2_). *R* ^AO^ (amplitude to offset ratio) observed in the first RSV of the SVD analysis are also shown for each time-series. Normalized data from this table are shown in Fig. 4.

	Short time-series											
	1	2	3	4	5	6	7	8	9	10	11	12
Average dose, uncorrected	3.3	6.6	9.9	13.2	16.5	19.8	23.1	26.4	29.7	33.0	36.3	39.6
(i) Dose corrected: crystal settings (*V*_C_ = 77%)	2.54	5.08	7.62	10.16	12.71	15.25	17.79	20.32	22.87	25.41	27.95	30.49
*y* (m)	0	0	0	0	2	5	8	11	12	13	14	16
(ii) Dose corrected (*D*_corr_): + edge scan	2.54	5.08	7.62	10.16	12.29	13.98	15.41	16.59	18.30	19.90	21.42	22.36
*N*_reflections_	3852	3860	3861	3861	3861	3859	3853	3864	3889	3908	3888	3894
*I*_32ms_	330	297	271	237	211	185	160	151	123	110	88	84
*I*_free_	337	310	289	258	233	206	181	172	142	128	104	100
*I*/_*I*__32ms_	11.07	10.22	9.35	8.23	7.33	6.50	5.71	5.31	4.42	3.98	3.33	3.26
*I*/_*I*__free_	11.31	10.67	9.97	9.00	8.10	7.28	6.47	6.07	5.12	4.66	3.94	3.89
_RSV_ (ms)	22	20	26	30	24	21	29	35	27	25	18	24
1/*k*_2_ (_post_) (ms)	23	23	27	30	48	65	95	89	168	136	113	nd
*R* ^AO^	6.04	7.27	5.72	6.12	3.91	3.04	2.49	2.45	2.07	1.8	1.57	1.53

**Table 2:** *D*_1/2_ and 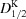 derived from uncorrected and corrected data The dose was corrected by the common volume and the extent of vertical crystal translation. X-ray intensities and *I*/_*I*_ were corrected in addition to account for the laser damage.

	*D*_1/2_	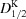
*I*, uncorrected	18.4 10^5^Gy	
*I*/_*I*_, uncorrected	19.7 10^5^Gy	
*I*_free_, corrected	16.8 10^5^Gy	
*I*/_*I*__free_, corrected	16.8 10^5^Gy	
*R* ^AO^, dose uncorrected†		16.0 10^5^Gy
*R* ^AO^, dose corrected		9.6 10^5^Gy

† Not shown in Fig. 4 ▶.

## Footnotes

1 Supplementary data for this paper are available from the IUCr electronic archives (Reference: RX5003). Services for accessing these data are described at the back of the journal.
