# Supplementary material for: The kinetic dose limit in room-temperature time-resolved macromolecular crystallography
Source: J Synchrotron Radiat. 2012 Jan 18;19(Pt 2):264–73. doi: 10.1107/S090904951105549X (PMC3284346; doi:10.1107/S090904951105549X)
Supplement: Supplementary file 1 [file s-19-00264-sup1.pdf]

Supplemental Material

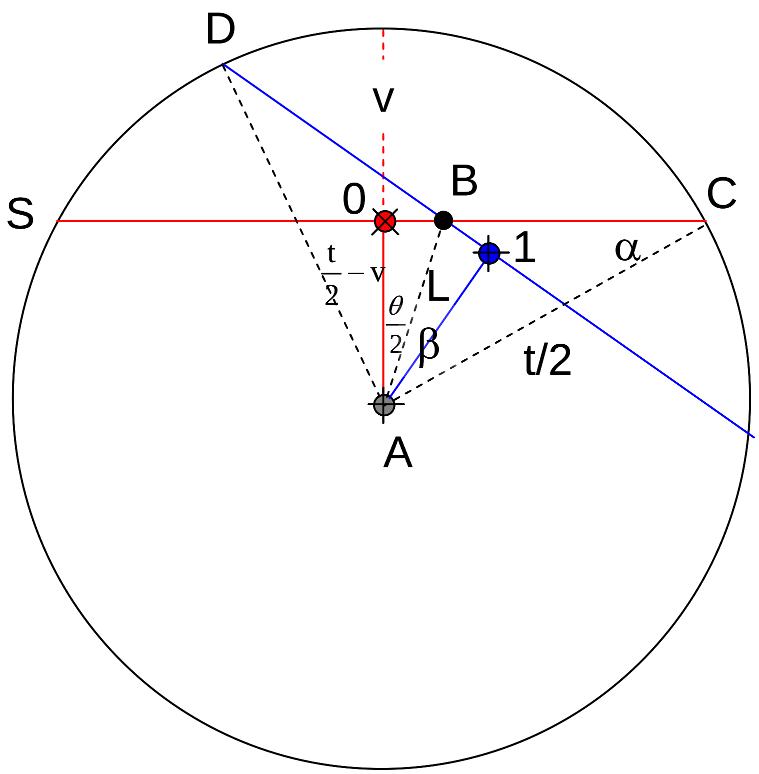

Figure S1. Scheme of the effect of an orientational change of a crystal of thickness t on the common volume. The radius of the circle is t/2 (see line AC). The vertical X-ray beam size v is smaller than t/2, hence the beam is offset a distance $t/2-\mathrm{v}$ from the center. The crystal is reoriented by an angle θ that moves the normal on the chord from position 0 to position 1. L bisects the angle θ, resulting in a rectangular triangle A0B with the angle θ/2 as shown. The task is to determine the area of the circular wedge DBC and relate it to the circular segment lined by the chord SC.

Calculation of the common volume

The circular segment DBC denotes the common area. The relative common volume in percent is the ratio of the segment DBC relative to the area of a circular cap (segment) bounded by the chord SC times 100. The area A of the segment bounded by the chord SC can be calculated as

$$A=\left(\frac{t}{2}\right)^2 \arccos\left(1-\frac{2\mathrm{v}}{\mathrm{t}}\right)-\sqrt{t\mathrm{v}-\mathrm{v}^2}\left(\frac{t}{2}-\mathrm{v}\right). \qquad \text{(S1)}$$

The calculation of the segment DBC is more difficult. If we rotate an angle θ, the normal on the chord rotates from position 0 to position 1 (see drawing). The line AB (denoted L) bisects the angle θ. We can calculate L as

$$L = \frac{\left(\frac{t}{2} - \mathrm{v}\right)}{\cos\frac{\theta}{2}}. \quad \text{(S2)}$$

We then determine the angle 0BA, which is: $\angle 0BA = 90^o - \frac{\theta}{2}$. From this we can determine the angle ABC: $\angle ABC = 90^o + \frac{\theta}{2}$. Using the rectangular triangle A0C, we can determine the angle α as: $\alpha = \arcsin\left(1 - \frac{2\mathrm{v}}{t}\right)$. Since we have now determined two of the three angles of triangle ABC, we can determine the last, β as:

$$\beta = 180^o - \angle ABC - \alpha = 90^0 - \frac{\theta}{2} - \arcsin\left(1 - \frac{2\mathrm{v}}{\mathrm{t}}\right). \quad \text{(S3)}$$

The angle β is also referred to in the text. Since triangle ABD is obtained from ABC by mirroring it at L, both triangles share the same angle β, and consequently 2β is the opening angle of the circular sector ACD, whose area can be easily calculated:

$$F_s = \frac{1}{2}\frac{2\beta}{180^o}\pi\left(\frac{t}{2}\right)^2. \quad \text{(S4)}$$

In order to calculate the area of the section DBC we need to subtract from F_s the two triangles ABC and ABD whose areas are the same. We have determined two sides and all angles of the triangle ABC, and its area is consequently:

$$F_T = \frac{1}{2}\frac{t}{2}L\sin\beta = \frac{t}{4}\frac{\left(\frac{t}{2} - \mathrm{v}\right)}{\cos\frac{\theta}{2}}\sin\beta. \quad \text{(S5)}$$

So the common area is now:

$$F_C = F_S - 2F_T = \frac{1}{2}\frac{2\beta}{180^o}\pi\left(\frac{t}{2}\right)^2 - \frac{t}{2}\frac{\left(\frac{t}{2} - \mathrm{v}\right)}{\cos\frac{\theta}{2}}\sin\beta. \quad \text{(S6)}$$

Equation S6 is referenced in the text as eqn. 1 with the first term slightly simplified and t/2 factored out of the bracket in the second term (see also eqn. S7), and it is also shown in fig. 2B.
The common volume V_C in percent is $F_C / A \times 100$:

$$V_C[\%] = \frac{\left(\frac{t}{2}\right)^2 \left[\frac{\beta}{180^o}\pi - \frac{\left(1-\frac{2\mathrm{v}}{\mathrm{t}}\right)}{\cos\frac{\theta}{2}} \sin\beta \right]}{\left(\frac{t}{2}\right)^2 \arccos\left(1-\frac{2\mathrm{v}}{\mathrm{t}}\right) - \sqrt{t\mathrm{v} - \mathrm{v}^2}\left(\frac{t}{2} - \mathrm{v}\right)} \times 100 \,. \qquad \text{(S7)}$$

Equation S7 was implemented in a Fortran program and its average was determined using all 5 membered-neighbors that are common to one crystal setting. The dose was corrected by this average.

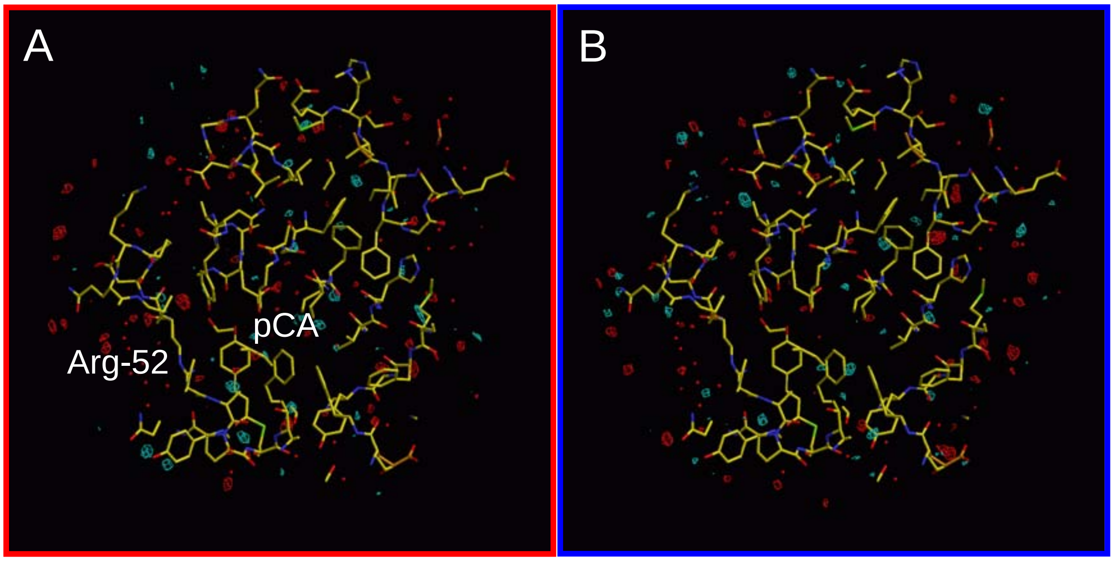

Figure S2. Difference maps overlaid on the dark structure of PYP. Panel A: Difference map obtained from the dark data of short time-series 1 and 12 (D12-D1 difference map) contoured on the +/-3 sigma level (cyan and red, respectively). Panel B: Difference maps obtained from the laser control experiment (D5-D1 difference map). Contour levels as in A.
